# *Salmonella* Typhimurium in Hihi, New Zealand

**DOI:** 10.3201/eid1305.060824

**Published:** 2007-05

**Authors:** John G. Ewen, Rose Thorogood, Carolyn Nicol, Doug P. Armstrong, Maurice Alley

**Affiliations:** * Institute of Zoology, Zoological Society of London, London, United Kingdom; † University of Cambridge, Cambridge, United Kingdom; ‡ Institute of Environmental Science and Research, Porirua, Wellington, New Zealand; § Massey University, Palmerston North, New Zealand

**Keywords:** Salmonella enterica serotype Typhimurium, Notiomystis cinct, hihi, birds, New Zealand, letter

**To the Editor:** The recent finding of a previously unrecorded *Salmonella* strain in an endangered New Zealand passerine (the hihi, *Notiomystis cincta*; [*1*]) offers the rare opportunity to observe the initial arrival and pathology of an epizootic and to determine its population-level effect. Over 8 days in February 2006, 6 freshly dead hihi were discovered in a free-living island population. Pathologic findings were similar: birds were in good body condition with substantial subcutaneous fat reserves and no gross lesions in the crop, indicating death from a highly pathogenic disease. Histopathologic examination showed septicemia and inflammatory necrosis of organs, particularly the liver and spleen, typical of salmonellosis in birds (*2*)*.* Microbiologic examination of liver samples isolated heavy growths of the bacterium *Salmonella enterica* serotype Typhimurium DT195. During the same period, 3 more dead hihi were found, but they were too decomposed for postmortem examination.

Hihi are nectar-feeders that declined to near extinction after European colonization of New Zealand and survived on a single island refuge (Hauturu). Since 1980, 14 attempts have been made to reintroduce the species to 6 other sites, resulting in 3 new populations that persist with management. The *S.* Typhimurium DT195 outbreak occurred within a reintroduced population on Tiritiri Matangi Island. Management includes providing supplementary food (sugar water) diluted with local rain water; feeders are sterilized before each use.

Because disease in hihi is closely monitored, the outbreak indicates that *S.* Typhimurium DT195 is a novel serotype for this species. During December 2005, fecal screening of 18 broods (37 nestlings) from Tiritiri Matagni Island found no evidence of enteric pathogens; screenings in February and May 2005 (40 adult and juvenile birds) from Tiritiri Matagni Island similarly returned negative results. Screening in all hihi populations during 2004 also found no evidence of *Salmonella* infection (32 adults and juveniles at Tiritiri Matangi, 29 at Hauturu, and 27 at Kapiti), and a 15-year pathology database from 230 dead hihi collected across these populations and a captive breeding facility lists no salmonellosis cases (J.G. Ewen and M.R. Alley, unpub. data).

Documentation of the emergent stages of infectious disease in endangered species is rare (*3*,*4*). This bacterium strain is absent from New Zealand’s livestock and wildlife (www.surv.esr.cri.nz/enteric_reference/nonhuman_salmonella.php). Nontyphoid *Salmonella* spp. are a major health concern worldwide (*5*), and New Zealand conducts intensive surveillance to maintain food safety. The New Zealand Wildlife Health Centre has not reported *S.* Typhimurium DT195 despite necropsies of >3,000 wild birds during 1996–2006, which suggests this strain is rare in New Zealand, despite its presence in other countries (*6*).

*S.* Typhimurium DT195 has been detected in 3 human patients in New Zealand (1 each in 2002, 2003, and 2006). The *S.* Typhimurium DT195 isolated from hihi in the February 2006 outbreak were indistinguishable from those isolated from the human case-patient in 2006 (Figure, panel A) (*2*). Tiritiri Matangi is an isolated island nature reserve 3 km off the New Zealand coast, which prevents movement of hihi to other areas. How this strain appeared in a human patient and as an epizootic in an isolated island nature reserve is intriguing. The most recent human case was diagnosed on the North Island of New Zealand, but the person was not living in close proximity to the birds. Tiritiri Matangi receives ≈30,000 human visitors per year, but whether the person with *S.* Typhimurium DT195 ever visited is not known. An unidentified infection source may be present in New Zealand that periodically spills over into alternate host species. Given their historic isolation, hihi may have low or no exposure to many diseases, which makes negative reactions more likely (*7*).

The transmission of *S.* Typhimurium DT195 to hihi caused a substantial drop in their population (Figure). The 9 bodies recovered represent a small proportion of the birds that died, given the difficulty of recovering dead birds (*8*). We used mark–recapture analysis (*9*) to estimate that adult survival probability was 0.64 (95% confidence interval [CI] 0.53–0.74) from September 2005 through February 2006, compared with an expected survival of 0.87 (95% CI 0.85–0.89), according to data from the previous 10 years (data not shown). The quotient of these 2 probabilities is 0.74 (95% CI 0.60–0.84); hence, we can infer that ≈26% of birds were killed by the epizootic.

**Figure:**
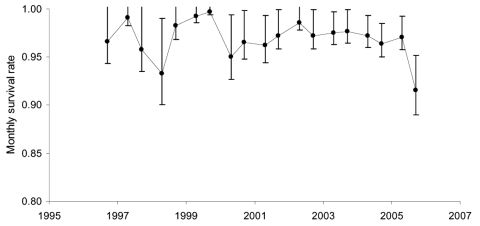
Survival rates from September–February and February–September among hihi in New Zealand during 1996–2006, estimated by using mark–recapture analysis, show that the transmission of *Salmonella* Typhimurium DT195 to hihi during the February 2006 epidemic caused a substantial drop in population. Bars indicate 95% confidence intervals.

With such high virulence, fade-out may occur as susceptible individuals are rapidly removed from the population (*10*). Subsequent monitoring has failed to detect further evidence of *S.* Typhimurium DT195. This apparent fade-out mirrors classic predictions from epidemiology (*10*). It is unknown whether the pathogen resides in resistant hihi or whether threats from the unknown source remain.

The key issues for endangered species management are identifying the risk of pathogens entering a host population and the probability that this occurrence would result in host extinction (*3*). The 2006 salmonellosis outbreak in hihi could easily have remained undetected, leaving conservation managers unaware of what caused the population decline. How often this occurs in poorly monitored wildlife is unknown. This study shows the need for increased awareness of these processes when considering biodiversity conservation.
